# Supplementary material for: Identification and Allele Combination Analysis of Rice Grain Shape-Related Genes by Genome-Wide Association Study
Source: Int J Mol Sci. 2022 Jan 19;23(3):1065. doi: 10.3390/ijms23031065 (PMC8835367; doi:10.3390/ijms23031065)
Supplement: Supplementary file 1 [file ijms-23-01065-s001.zip › ijms-1539899-supplementary.pdf]

Supplementary Material

1 Supplementary Tables

Supplementary Table S1.All QTLs

QTL	Env	Trait	CHRO	Position	Peak-SNP	Ref/Alt	Effect	SE	*P*	PVE(%)	Cloned gene
qGC2	2018GA	GC	2	34214439-34472981	chr02_34347981	G/A	-0.555	0.099	2.88316×10^{-8}	2.96	*OsmiR396a;OsmiR396c*
qGC3	2017GA	GC	3	16538239-17145970	chr03_16707611	T/C	-0.392	0.047	4.618×10^{-16}	42.07	*GS3*
	2017EZ	GC	3	16538239-17145970	chr03_16707611	T/C	-0.429	0.055	1.98333×10^{-14}	41.50	*GS3*
	2018GA	GC	3	16538239-17145970	chr03_16692834	A/G	-0.488	0.057	1.64375×10^{-16}	38.46	*GS3*
	2018EZ	GC	3	16538239-17145970	chr03_16669223	T/C	-0.518	0.060	4.16047×10^{-17}	36.18	*GS3*
qGC4	2017GA	GC	4	20291326-20541326	chr04_20416326	G/A	-0.341	0.064	1.18241×10^{-7}	4.02	
qGC5	2017GA	GC	5	5233756-5486894	chr05_5361894	G/A	-0.215	0.039	4.55499×10^{-8}	33.45	*GW5;OsDER1*
qGC10.1	2017GA	GC	10	981239-1231239	chr10_1106239	G/T	-0.417	0.076	6.05196×10^{-8}	7.84	
qGC10.2	2017GA	GC	10	9867724-10473419	chr10_10261032	C/T	-0.573	0.090	4.56979×10^{-10}	5.32	
qGC10.3	2017GA	GC	10	11658222-11908222	chr10_11783222	G/A	-0.480	0.085	2.31857×10^{-8}	7.46	
qGC10.4	2017GA	GC	10	16308002-16744439	chr10_16598284	T/G	-0.571	0.086	8.77068×10^{-11}	7.46	
	2018GA	GC	10	16473561-16723561	chr10_16598561	A/G	0.298	0.058	3.72151×10^{-7}	0.51	
qGL2	2018GA	GL	2	34202268-34492940	chr02_34339439	G/A	-0.239	0.044	9.10759×10^{-8}	4.34	*OsmiR396a;OsmiR396c*
qGL3.1	2017GA	GL	3	1-198308	chr03_25257	C/T	-0.261	0.046	2.10915×10^{-8}	7.91	
qGL3.2	2018GA	GL	3	16121544-16371544	chr03_16246544	G/A	0.110	0.020	9.6657×10^{-8}	20.37	
qGL3.3	2017EZ	GL	3	16538239-17145970	chr03_16692706	C/T	-0.215	0.028	3.9741×10^{-14}	41.52	*GS3*
	2018GA	GL	3	16538239-17145970	chr03_16692834	A/G	-0.236	0.027	7.18544×10^{-14}	40.03	*GS3*
	2017GA	GL	3	16538239-17145970	chr03_16727804	G/A	-0.187	0.023	1.00333×10^{-15}	39.81	*GS3*
	2018EZ	GL	3	16538239-17145970	chr03_16669223	T/C	-0.242	0.029	3.25938×10^{-16}	35.72	*GS3*
qGL4.1	2017GA	GL	4	20291326-20541326	chr04_20416326	G/A	-0.170	0.032	1.64029×10^{-7}	4.91	
qGL4.2	2017GA	GL	4	20660911-21006646	chr04_20881646	G/A	-0.185	0.034	7.89811×10^{-8}	2.71	
	2017EZ	GL	4	20664142-20914142	chr04_20789142	A/C	-0.199	0.039	3.85431×10^{-7}	1.62	
qGL5	2017GA	GL	5	5233629-5496042	chr05_5361894	G/A	-0.132	0.020	3.72538×10^{-11}	35.53	*GW5;OsDER1*
	2017EZ	GL	5	5233629-5496042	chr05_5361894	G/A	-0.145	0.024	1.49047×10^{-9}	34.90	*GW5;OsDER1*
	2018EZ	GL	5	5233629-5488611	chr05_5359598	G/A	-0.138	0.026	9.59975×10^{-8}	24.92	*GW5;OsDER1*
	2018GA	GL	5	5234598-5484598	chr05_5359598	G/A	-0.121	0.024	4.07439×10^{-7}	27.56	*GW5;OsDER1*
qGL10.1	2017GA	GL	10	981239-1231239	chr10_1106239	G/T	-0.203	0.039	1.9934×10^{-7}	9.09	
qGL10.2	2017GA	GL	10	4487058-4737058	chr10_4612058	T/G	-0.193	0.038	3.77347×10^{-7}	9.25	
qGL10.3	2017GA	GL	10	9863348-10473419	chr10_10261032	C/T	-0.301	0.045	8.07794×10^{-11}	6.75	
qGL10.4	2017GA	GL	10	11658222-11908222	chr10_11783222	G/A	-0.238	0.042	2.86444×10^{-8}	9.00	
qGL10.5	2017GA	GL	10	16308002-16744439	chr10_16598415	T/A	-0.279	0.043	2.88314×10^{-10}	8.89	
qGL12	2017GA	GL	12	22015686-22422502	chr12_22157504	C/G	0.184	0.033	3.96041×10^{-18}	1.85	
qLWR1.1	2017GA	GLWR	1	3182916-3432916	chr01_3307916	A/G	0.222	0.039	1.93411×10^{-8}	0.57	
	2018EZ	GLWR	1	3182916-3432916	chr01_3307916	A/G	0.214	0.041	2.85427×10^{-7}	0.38	
	2017EZ	GLWR	1	3182916-3432916	chr01_3307916	A/G	0.213	0.040	1.86665×10^{-7}	0.32	
qLWR1.2	2018EZ	GLWR	1	22885450-23135450	chr01_23010450	A/G	-0.287	0.052	4.5957×10^{-8}	9.95	

	2018GA	GLWR	1	22885450-23135450	chr01_23010450	A/G	-0.276	0.052	1.61289×10^{-7}	9.36	
	2017EZ	GLWR	1	22885450-23137896	chr01_23010450	A/G	-0.301	0.051	5.90519×10^{-9}	9.03	
qLWR1.3	2017EZ	GLWR	1	23971227-24526872	chr01_24396533	G/T	-0.312	0.058	8.44178×10^{-8}	7.09	
	2018GA	GLWR	1	24214872-24464872	chr01_24339872	A/G	-0.316	0.060	1.8032×10^{-7}	7.02	
qLWR2.1	2018EZ	GLWR	2	3328503-3578511	chr02_3453511	C/A	-0.180	0.035	2.76336×10^{-7}	7.68	
	2017EZ	GLWR	2	3328503-3578581	chr02_3453511	C/A	-0.192	0.034	2.33924×10^{-8}	7.39	
	2018GA	GLWR	2	3328511-3578511	chr02_3453511	C/A	-0.179	0.035	3.7586×10^{-7}	7.16	
qLWR2.2	2017GA	GLWR	2	5535710-5785710	chr02_5660710	T/A	-0.330	0.056	5.91233×10^{-9}	6.60	
	2018GA	GLWR	2	5535710-5999873	chr02_5660710	T/A	-0.324	0.060	8.1989×10^{-8}	6.34	
	2018EZ	GLWR	2	5535710-5999873	chr02_5874873	C/T	-0.373	0.071	2.23701×10^{-7}	6.08	
	2017EZ	GLWR	2	5535710-6323430	chr02_5660710	T/A	-0.347	0.057	2.70846×10^{-9}	5.92	
	2018EZ	GLWR	2	6073121-6323430	chr02_6198419	G/T	-0.338	0.064	1.90726×10^{-7}	6.22	
	2018GA	GLWR	2	6073121-6323430	chr02_6198419	G/T	-0.358	0.065	4.58012×10^{-8}	5.76	
qLWR2.3	2017EZ	GLWR	2	11224753-11483573	chr02_11358094	C/T	0.257	0.049	2.63321×10^{-7}	3.37	
	2018GA	GLWR	2	11233094-11483573	chr02_11358094	C/T	0.257	0.050	3.51701×10^{-7}	3.35	
qLWR2.4	2017GA	GLWR	2	12281126-12531126	chr02_12406126	C/T	-0.313	0.058	1.20528×10^{-7}	11.47	
qLWR2.5	2018EZ	GLWR	2	12581170-12854381	chr02_12706170	G/A	-0.375	0.067	3.83638×10^{-8}	12.35	
	2017GA	GLWR	2	12581170-12854381	chr02_12706170	G/A	-0.356	0.065	5.90344×10^{-8}	12.21	
	2018GA	GLWR	2	12581170-12831170	chr02_12706170	G/A	-0.367	0.068	1.02856×10^{-7}	11.60	
	2017EZ	GLWR	2	12581170-12854381	chr02_12729381	C/T	-0.377	0.067	3.44126×10^{-8}	10.28	
qLWR2.6	2018EZ	GLWR	2	13775664-14025664	chr02_13900664	A/G	-0.341	0.066	3.74571×10^{-7}	11.84	
qLWR2.7	2017GA	GLWR	2	15065202-15315202	chr02_15190202	C/T	-0.253	0.049	2.83929×10^{-7}	10.74	
qLWR3.1	2017GA	GLWR	3	11862123-12195220	chr03_12040893	G/A	-0.238	0.042	2.0198×10^{-8}	10.29	
	2017EZ	GLWR	3	11862123-12180797	chr03_12040893	G/A	-0.226	0.043	1.76231×10^{-7}	9.85	
qLWR3.2	2017GA	GLWR	3	14887077-15259083	chr03_15097804	T/C	-0.379	0.066	1.73085×10^{-8}	11.94	
	2018EZ	GLWR	3	14972804-15262319	chr03_15137319	G/C	-0.370	0.063	8.16489×10^{-9}	10.84	
	2017EZ	GLWR	3	14972804-15775796	chr03_15137319	G/C	-0.377	0.062	1.86381×10^{-9}	9.87	
	2018GA	GLWR	3	15007020-15499312	chr03_15137319	G/C	-0.377	0.064	6.37505×10^{-9}	10.27	
	2018GA	GLWR	3	15525398-15798206	chr03_15650795	C/T	-0.261	0.048	8.10411×10^{-8}	9.26	
	2018EZ	GLWR	3	15525795-15798182	chr03_15673182	T/A	-0.253	0.049	2.89611×10^{-7}	9.09	
qLWR3.3	2018EZ	GLWR	3	16384313-17145970	chr03_16665078	G/A	-0.253	0.030	5.54904×10^{-16}	38.74	*GS3*
	2017EZ	GLWR	3	16538239-17145970	chr03_16667236	A/C	-0.247	0.030	4.99029×10^{-16}	40.92	*GS3*
	2017GA	GLWR	3	16538239-17144509	chr03_16665078	G/A	-0.242	0.030	1.84033×10^{-15}	38.89	*GS3*
	2018GA	GLWR	3	16538239-17145970	chr03_16665078	G/A	-0.249	0.031	2.87413×10^{-15}	38.73	*GS3*
qLWR4.1	2017EZ	GLWR	4	11113-261113	chr04_136113	G/A	-0.255	0.050	3.91553×10^{-7}	7.94	
	2018GA	GLWR	4	11113-261113	chr04_136113	G/A	-0.268	0.051	1.65869×10^{-7}	7.75	
qLWR4.2	2017EZ	GLWR	4	274309-524309	chr04_399309	A/G	-0.268	0.051	1.84523×10^{-7}	7.28	*OsARG*
qLWR4.3	2017EZ	GLWR	4	550893-800893	chr04_675893	C/T	-0.293	0.056	2.11115×10^{-7}	7.57	
qLWR4.4	2018EZ	GLWR	4	20288335-20541326	chr04_20416326	G/A	-0.229	0.041	3.2422×10^{-8}	7.48	

	2017EZ	GLWR	4	20288335-21006646	chr04_20416326	G/A	-0.226	0.040	2.42506×10^{-8}	6.86	
	2018GA	GLWR	4	20291326-20541326	chr04_20416326	G/A	-0.220	0.041	1.22254×10^{-7}	6.94	
	2018EZ	GLWR	4	20593177-21006646	chr04_20718177	G/A	-0.215	0.041	2.54712×10^{-7}	5.45	
	2017GA	GLWR	4	20638861-21006958	chr04_20881646	G/A	-0.254	0.042	3.01979×10^{-9}	5.45	
qLWR5.1	2018EZ	GLWR	5	2033721-2285343	chr05_2158721	G/A	0.211	0.041	3.41838×10^{-7}	1.40	
	2017EZ	GLWR	5	2034040-2284040	chr05_2159040	C/T	0.207	0.040	4.13282×10^{-7}	1.79	
qLWR5.2	2017EZ	GLWR	5	5231448-5561924	chr05_5361894	G/A	-0.214	0.025	5.32542×10^{-17}	42.53	*GW5;OsDER1*
	2018GA	GLWR	5	5231448-5561924	chr05_5361894	G/A	-0.231	0.025	1.41282×10^{-18}	41.88	*GW5;OsDER1*
	2017GA	GLWR	5	5231448-5503981	chr05_5359598	G/A	-0.210	0.024	5.38154×10^{-17}	41.49	*GW5;OsDER1*
	2018EZ	GLWR	5	5231448-5561924	chr05_5359598	G/A	-0.232	0.025	1.19804×10^{-18}	40.73	*GW5;OsDER1*
qLWR7.1	2017EZ	GLWR	7	22407359-22771306	chr07_22646306	C/T	-0.265	0.050	2.10854×10^{-7}	5.63	
	2017GA	GLWR	7	22509519-22759519	chr07_22634519	C/T	-0.253	0.048	2.27179×10^{-7}	6.53	
qLWR7.2	2017GA	GLWR	7	23874546-24124546	chr07_23999546	G/T	-0.273	0.051	1.32149×10^{-7}	10.23	
	2017EZ	GLWR	7	23874546-24124546	chr07_23999546	G/T	-0.275	0.053	2.92081×10^{-7}	9.27	
qGLWR10.1	2018EZ	GLWR	10	461202-711202	chr10_586202	A/T	-0.241	0.045	1.11028×10^{-7}	12.01	
	2018GA	GLWR	10	461202-711202	chr10_586202	A/T	-0.237	0.045	1.8842×10^{-7}	11.28	
	2017EZ	GLWR	10	461202-711202	chr10_586202	A/T	-0.253	0.045	4.17056×10^{-8}	10.75	
qGLWR10.2	2017GA	GLWR	10	981239-1231239	chr10_1106239	G/T	-0.251	0.049	3.35516×10^{-7}	10.74	
qGLWR10.3	2017GA	GLWR	10	1931648-2181648	chr10_2056648	C/A	0.260	0.051	3.72935×10^{-7}	0.18	
qGLWR10.4	2017GA	GLWR	10	3451996-3701996	chr10_3576996	G/A	-0.256	0.048	1.5324×10^{-7}	10.03	
qGLWR10.5	2017EZ	GLWR	10	4480128-4738419	chr10_4612011	A/G	-0.276	0.046	2.50401×10^{-9}	11.20	
	2017GA	GLWR	10	4487011-4737058	chr10_4612058	T/G	-0.283	0.046	1.62479×10^{-9}	11.26	
	2018EZ	GLWR	10	4487011-4737058	chr10_4612058	T/G	-0.254	0.048	2.08188×10^{-7}	10.79	
	2018GA	GLWR	10	4487011-4737058	chr10_4612058	T/G	-0.263	0.049	9.96257×10^{-8}	10.62	
qGLWR10.6	2018EZ	GLWR	10	5900422-6150495	chr10_6025464	G/A	-0.304	0.053	1.57353×10^{-8}	9.73	
	2017EZ	GLWR	10	5900422-6150524	chr10_6025464	G/A	-0.346	0.052	5.39359×10^{-11}	9.46	
	2018GA	GLWR	10	5900422-6150495	chr10_6025464	G/A	-0.315	0.053	5.45461×10^{-9}	9.25	
qGLWR10.7	2018EZ	GLWR	10	7593442-7843442	chr10_7718442	T/C	-0.313	0.058	8.71587×10^{-8}	9.06	
	2018GA	GLWR	10	7593442-7843442	chr10_7718442	T/C	-0.311	0.058	1.33752×10^{-7}	8.50	
	2017EZ	GLWR	10	7593442-7843442	chr10_7718442	T/C	-0.321	0.056	1.63274×10^{-8}	8.11	
qGLWR10.8	2018EZ	GLWR	10	8856195-9106195	chr10_8981195	G/T	-0.332	0.062	1.05397×10^{-7}	10.16	
	2018GA	GLWR	10	8856195-9106195	chr10_8981195	G/T	-0.332	0.062	1.42668×10^{-7}	9.51	
	2017EZ	GLWR	10	8856195-9106195	chr10_8981195	G/T	-0.336	0.060	3.12832×10^{-8}	9.06	
qGLWR10.9	2017EZ	GLWR	10	10025822-10458283	chr10_10223273	C/T	-0.381	0.059	2.51359×10^{-10}	8.23	
	2017GA	GLWR	10	10042380-10458283	chr10_10272029	G/A	-0.363	0.061	4.73434×10^{-9}	9.43	
	2018EZ	GLWR	10	10042380-10397029	chr10_10223273	C/T	-0.363	0.060	2.98474×10^{-9}	9.16	
	2018GA	GLWR	10	10042380-10397029	chr10_10223273	C/T	-0.372	0.061	1.97669×10^{-9}	8.79	
qGLWR10.10	2017GA	GLWR	10	11431732-11681732	chr10_11556732	G/A	-0.256	0.049	2.32483×10^{-7}	9.72	
	2017EZ	GLWR	10	11575655-11825757	chr10_11700757	G/A	-0.343	0.063	6.82111×10^{-8}	6.94	
qGLWR10.11	2018EZ	GLWR	10	18701773-18951773	chr10_18826773	G/A	-0.311	0.061	4.09216×10^{-7}	7.75	

	2017EZ	GLWR	10	18701773-18951780	chr10_18826773	G/A	-0.320	0.059	8.0211×10^{-8}	6.46	
qGLWR10.12	2017EZ	GLWR	10	20758834-21008834	chr10_20883834	G/A	0.281	0.053	1.98776×10^{-7}	0.05	
qGLWR12	2017GA	GLWR	12	22008768-22432790	chr12_22146065	G/T	0.218	0.038	2.07391×10^{-8}	1.39	
qGS3	2018EZ	GS	3	22314143-22564143	chr03_22439143	C/T	0.536	0.101	1.38297×10^{-7}	2.41	
qGS8	2018GA	GS	8	16039613-16289613	chr08_16164613	G/C	0.502	0.098	3.73176×10^{-7}	0.50	
qGW1.1	2018GA	GW	1	3172836-3498025	chr01_3373022	G/A	-0.086	0.016	1.93132×10^{-7}	0.31	
qGW1.2	2017EZ	GW	1	22887896-23137896	chr01_23012896	G/A	0.101	0.019	1.13218×10^{-7}	9.86	
qGW1.3	2018EZ	GW	1	23933241-24512660	chr01_24304654	A/G	0.113	0.022	2.42535×10^{-7}	6.23	*OsCTPS1*
qGW2.1	2017EZ	GW	2	3328503-3578511	chr02_3453511	C/A	0.069	0.012	1.13817×10^{-8}	6.07	
	2018EZ	GW	2	3328503-3578511	chr02_3453511	C/A	0.065	0.012	2.15744×10^{-7}	4.81	
	2018GA	GW	2	3328503-3578511	chr02_3453511	C/A	0.067	0.013	2.35387×10^{-7}	4.12	
qGW2.2	2018GA	GW	2	5535710-5785710	chr02_5660710	T/A	0.132	0.022	2.13262×10^{-9}	5.96	
qGW2.3	2018GA	GW	2	5912625-6323430	chr02_6198419	G/T	0.126	0.024	1.47254×10^{-7}	4.97	
qGW2.4	2017GA	GW	2	32515966-32765966	chr02_32640966	T/C	0.076	0.014	8.88731×10^{-8}	4.09	
qGW3.1	2017GA	GW	3	1-198308	chr03_53666	C/T	0.085	0.016	1.18065×10^{-7}	8.13	
qGW3.2	2017GA	GW	3	11862123-12165893	chr03_12040893	G/A	0.065	0.012	6.79508×10^{-8}	8.01	
qGW3.3	2018GA	GW	3	14752783-15262319	chr03_15137319	G/C	0.143	0.023	1.03012×10^{-9}	9.42	
qGW3.4	2017GA	GW	3	16538324-17028634	chr03_16706516	T/C	0.053	0.008	5.96404×10^{-11}	41.78	*GS3*
	2018EZ	GW	3	16540072-17040318	chr03_16706516	T/C	0.062	0.010	5.44229×10^{-10}	30.86	*GS3*
	2017EZ	GW	3	16540424-16926472	chr03_16706516	T/C	0.056	0.009	3.95865×10^{-9}	30.65	*GS3*
	2018GA	GW	3	16570517-16998965	chr03_16746142	A/G	0.059	0.011	2.73991×10^{-8}	25.80	*GS3*
qGW3.5	2017EZ	GW	3	20113546-20363546	chr03_20238546	T/G	0.093	0.018	3.98611×10^{-7}	5.98	
qGW4.1	2018GA	GW	4	11108-800893	chr04_628540	C/A	0.111	0.019	1.47095×10^{-8}	8.52	*OsARG*
	2017EZ	GW	4	11113-261113	chr04_136113	G/A	0.091	0.017	1.77411×10^{-7}	8.98	
	2018EZ	GW	4	263820-769400	chr04_644395	A/G	0.108	0.019	2.30805×10^{-8}	7.48	*OsARG*
	2017EZ	GW	4	546523-796523	chr04_671523	C/T	0.099	0.019	3.60601×10^{-7}	9.04	*OsARG*
qGW4.2	2018EZ	GW	4	838813-1088813	chr04_963813	C/T	0.096	0.017	6.57378×10^{-8}	7.44	
qGW4.3	2017EZ	GW	4	20494104-20744104	chr04_20619104	C/T	0.099	0.019	3.10045×10^{-7}	8.12	
	2018EZ	GW	4	20494104-20864422	chr04_20739422	G/T	0.113	0.019	3.00921×10^{-9}	7.07	
qGW5.1	2017GA	GW	5	5231448-5561924	chr05_5359246	G/A	0.065	0.007	2.13646×10^{-20}	42.12	*GW5;OsDER1*
	2018GA	GW	5	5231448-5585712	chr05_5359246	G/A	0.091	0.009	7.09101×10^{-22}	36.43	*GW5;OsDER1*
	2018EZ	GW	5	5231448-5574689	chr05_5359681	C/T	0.082	0.009	5.765×10^{-19}	36.22	*GW5;OsDER1*
	2017EZ	GW	5	5231448-5581997	chr05_5359246	G/A	0.071	0.008	7.27903×10^{-17}	31.13	*GW5;OsDER1*
qGW5.2	2018GA	GW	5	5917480-6167480	chr05_6042480	C/T	0.043	0.008	3.08835×10^{-7}	12.62	*JMJ703*
qGW5.3	2018EZ	GW	5	26193638-26443638	chr05_26318638	G/A	0.163	0.030	1.25009×10^{-7}	9.36	
qGW5.4	2018EZ	GW	5	28514404-28764404	chr05_28639404	C/T	0.102	0.019	1.68027×10^{-7}	6.42	
qGW6	2017GA	GW	6	9195926-9445926	chr06_9320926	T/C	0.086	0.016	1.87142×10^{-7}	4.81	
qGW9	2017GA	GW	9	10772253-11022253	chr09_10897253	C/T	0.056	0.011	3.7008×10^{-7}	8.47	
qGW10.1	2018GA	GW	10	52079-302079	chr10_177079	T/A	0.124	0.022	4.17434×10^{-8}	8.97	*OsSCP46*
	2018EZ	GW	10	52079-302079	chr10_177079	T/A	0.120	0.022	5.35845×10^{-8}	8.72	*OsSCP46*
qGW10.2	2018EZ	GW	10	325563-711202	chr10_450563	A/G	0.070	0.013	8.87036×10^{-8}	8.35	
	2017GA	GW	10	481907-731908	chr10_606907	C/T	0.098	0.017	2.1078×10^{-8}	8.33	
qGW10.3	2017GA	GW	10	1060479-1310479	chr10_1185479	T/C	-0.094	0.018	3.71558×10^{-7}	1.08	
qGW10.4	2017GA	GW	10	3451996-3701996	chr10_3576996	G/A	0.083	0.015	3.83749×10^{-8}	8.13	

qGW10.5	2017GA	GW	10	4155253-4405253	chr10_4280253	T/C	0.083	0.016	2.46942×10^{-7}	9.66
qGW10.6	2018GA	GW	10	4480458-4790777	chr10_4607877	C/T	0.087	0.016	1.79628×10^{-7}	9.62
	2017GA	GW	10	4480458-4779354	chr10_4612058	T/G	0.085	0.014	5.20266×10^{-9}	9.31
qGW10.7	2017EZ	GW	10	5900422-6150495	chr10_6025464	G/A	0.126	0.019	1.49782×10^{-10}	10.32
	2018GA	GW	10	5900422-6150524	chr10_6025464	G/A	0.142	0.021	7.97234×10^{-11}	8.94
	2018EZ	GW	10	5900464-6150464	chr10_6025464	G/A	0.112	0.022	3.22894×10^{-7}	7.82
qGW10.8	2017EZ	GW	10	6986882-7439539	chr10_7297872	A/C	0.121	0.020	4.16284×10^{-9}	10.05
	2018GA	GW	10	7154870-7422872	chr10_7279870	C/G	0.108	0.018	2.7638×10^{-9}	11.54
qGW10.9	2017EZ	GW	10	7503127-7843442	chr10_7718442	T/C	0.134	0.022	1.16937×10^{-9}	9.64
qGW10.10	2017EZ	GW	10	8427980-8678013	chr10_8553013	A/C	0.103	0.019	1.48095×10^{-7}	9.21
qGW10.11	2017EZ	GW	10	8854791-9106195	chr10_8981195	G/T	0.109	0.021	1.61204×10^{-7}	9.06
qGW10.12	2017EZ	GW	10	10078190-10459267	chr10_10223273	C/T	0.122	0.021	9.81178×10^{-9}	8.84
	2018EZ	GW	10	10078190-10405532	chr10_10203190	A/G	0.127	0.022	1.04852×10^{-8}	7.51
	2017GA	GW	10	10147029-10418094	chr10_10272029	G/A	0.095	0.018	1.02675×10^{-7}	7.89
qGW10.13	2018GA	GW	10	11575699-11825757	chr10_11700699	T/A	0.130	0.023	3.76398×10^{-8}	7.22
qGW10.14	2018EZ	GW	10	11872917-12122917	chr10_11997917	T/C	0.131	0.024	6.32019×10^{-8}	8.37
qGW10.15	2018GA	GW	10	20758834-21015012	chr10_20883834	G/A	-0.111	0.019	1.65359×10^{-8}	1.15
qGW11	2018GA	GW	11	24152293-24402293	chr11_24277293	C/T	-0.097	0.017	2.73615×10^{-8}	1.42
qGW12	2017GA	GW	12	22021065-22432790	chr12_22307790	G/C	-0.067	0.012	3.45525×10^{-8}	1.80
	2018GA	GW	12	22021065-22295855	chr12_22146065	G/T	-0.080	0.015	2.32514×10^{-7}	1.51

Supplementary Table S2. List of the 623 varieties used this study

Serial number	Variety name	Serial number	Variety name
Line 1	Chenghui448	Line 313	BATHURI
Line 2	Budda	Line 314	DALSUNG 41
Line 3	Minghui63	Line 315	DANGAR
Line 4	Chorofa	Line 316	B 6136-3-TB-0-1-5
Line 5	D11	Line 317	Zhongchao123
Line 6	D15	Line 318	Gang46B
Line 7	KR200	Line 319	CO 39
Line 8	829	Line 320	GRITNA
Line 9	MERLE	Line 321	JAGLI BORO
Line 10	RR 272-17-829	Line 322	Gaozi
Line 11	K 479-2-3	Line 323	BINUHANGIN
Line 12	Zaoxian14	Line 324	NX 3533
Line 13	IRGA 959-1-2-2F-4-1-4A-6-CA-6X	Line 325	Bg 94-1
Line 14	IR65600-27-1-2-2	Line 326	B6136-3-TB-0-1-5
Line 15	Zhongjian100	Line 327	BW311-9
Line 16	E 2040	Line 328	PSBRC80
Line 17	Guangluai4	Line 329	Adny 11
Line 18	Zhongyouzao81	Line 330	Chhomromg
Line 19	E ZI 96	Line 331	Palung 2
Line 20	IRGA 370-38-1-1F-C4-2	Line 332	FL478

Line 21	BHAINSA MUNDARIYA	Line 333	IR68
Line 22	IR 2344-P1 PB-9-3-2B	Line 334	SAGC－4
Line 23	Zhenshan97	Line 335	NPT-114
Line 24	ARROZVERMELHO	Line 336	Guangluai4
Line 25	117	Line 337	包协 123B
Line 26	MONOLAYA	Line 338	Zhongnong4
Line 27	OM997	Line 339	Liushizao
Line 28	LALSAITA	Line 340	N'YA NOFF
Line 29	IRGA 659-1-2-2-2	Line 341	Momi
Line 30	DANAU LAUT TAWAR	Line 342	Heidu4
Line 31	IR 57920-AC-25-2-B	Line 343	Bawangbian1
Line 32	WAS 173-B-B-6-2-2	Line 344	ARC 6579
Line 33	ARAURE 1	Line 345	INIAP 6
Line 34	AMISTAD 82	Line 346	Taizhong65
Line 35	ICTA CRISPO 38	Line 347	UQUIHUA
Line 36	IR 75870-5-8-5-B-1	Line 348	Qingsiai16B
Line 37	IR77298-14-1-2	Line 349	Menjiading2
Line 38	Bala	Line 350	Gu154
Line 39	IR55419-04	Line 351	CH1157
Line 40	Matatag2	Line 352	Ninghui21
Line 41	ARC 10100	Line 353	HONG DU BAI
Line 42	UNNAMED	Line 354	Fanhaopi
Line 43	Ajaya	Line 355	JHORA
Line 44	P59279	Line 356	SANTHI 206
Line 45	Guang122	Line 357	DONGREM
Line 46	IRAT144	Line 358	Aimakang
Line 47	93072	Line 359	R8006(Zhonghui8006)
Line 48	Maravilha	Line 360	IR8
Line 49	BG304	Line 361	IR36
Line 50	YZX1	Line 362	IR38（IR2070-423-2-5-6）
Line 51	42686	Line 363	IR64（IR18348-36-3-3）
Line 52	TKM 9	Line 364	IR6
Line 53	CHANDINA	Line 365	IR72
Line 54	YA NONG ZAO 4	Line 366	Minghui86
Line 55	YSBR1	Line 367	Kanghui63
Line 56	BD007	Line 368	Luhui63
Line 57	E 2024	Line 369	Ce49（Ce64-7-49）
Line 58	GLADIO	Line 370	Ce64
Line 59	Karnal Local	Line 371	Duohui1
Line 60	Y134	Line 372	Mianhui734
Line 61	Amol3(Sana)	Line 373	PC311
Line 62	Bg 300	Line 374	Wanhui88
Line 63	BEGMI 135	Line 375	R288
Line 64	YZX2	Line 376	6078 (Yuhui6078）
Line 65	IR64a	Line 377	Shengtai1

Line 66	CALIFORNIA BELLE	Line 378	Hua-2
Line 67	WAS 206-B-B-2-2-1	Line 379	Manghui
Line 68	WAS 198-B-3-1-3	Line 380	Zhenhui084
Line 69	IR 56	Line 381	R287
Line 70	IR8192-200-3-3-1-1	Line 382	Chenghui178
Line 71	BA SHI ZAO	Line 383	Enhui69
Line 72	Y075	Line 384	R1128
Line 73	NERICA-L-27	Line 385	II-32B
Line 74	Molizhanxuan	Line 386	Jin23B
Line 75	Wanxian763	Line 387	WuxiangB
Line 76	CT 6510-24-1-2	Line 388	V20B
Line 77	SONA(IET 1991)	Line 389	BoB
Line 78	Fengaizhan	Line 390	MaxieB
Line 79	Yuexiangzhan	Line 391	CTB
Line 80	Hnankar	Line 392	898B
Line 81	IR68552-55-3-2	Line 393	05CR89B
Line 82	OM1706	Line 394	EjinB
Line 83	IRBB7	Line 395	Ezao18
Line 84	IR64-IL	Line 396	Yue4B
Line 85	IR68897B	Line 397	Runzhu537（Ezhong5）
Line 86	PSBRC82	Line 398	Yuexiangzhan
Line 87	NERICA-L-1	Line 399	Xiangdao
Line 88	Huhan15	Line 400	Molixiangzhan
Line 89	Giza14	Line 401	Gaodanbaixiangdao
Line 90	UPL RI-7	Line 402	Jianzhen2
Line 91	B 6144-MR-6-0-0	Line 403	BG304
Line 92	GIZA 178	Line 404	Bataixiangzhan
Line 93	SAMBALA MALO	Line 405	Huhan3
Line 94	WAS 199-B-1-2-1	Line 406	Yangdao4
Line 95	IRI 339	Line 407	Milyang83（Miyang83）
Line 96	IR 57514-PMI 5-B-1-2	Line 408	Wenshengnuo
Line 97	Jiangxisimiao	Line 409	Ewan13
Line 98	X21	Line 410	Wen229
Line 99	C70	Line 411	Zaoyou143（ Xiangzao143）
Line 100	LX2007	Line 412	Yuanfengzao
Line 101	MAYBELLE	Line 413	Enhui58
Line 102	EX FOILAEIN(NAPUTO)	Line 414	Guanghui380
Line 103	IR 21015-72-3-3-3-1	Line 415	Guanghui880
Line 104	UPR 1201-1-20-1	Line 416	158B
Line 105	RPW9-4(SS1)	Line 417	814B （Jingchu814B）
Line 106	R644	Line 418	Miyang83
Line 107	Carijo	Line 419	Gunong2923
Line 108	IET1444	Line 420	Guojisuo1
Line 109	Ganwanxian37（926）	Line 421	Guooyou12
Line 110	Huangsizhan	Line 422	#02428

Line 111	IR 2003-P7-7-4-2	Line 423	Zhaiyeqing8
Line 112	SANHUANGZHAN NO 2	Line 424	Forbiprotife
Line 113	IR 80310-12-B-1-3-B	Line 425	Ezao6
Line 114	Bg90-2	Line 426	Ezao11
Line 115	Govind	Line 427	Xiangzaoxian21
Line 116	NSIC RC9 (APO)	Line 428	Zhenguiai
Line 117	Hua565	Line 429	ShijinB
Line 118	PT60	Line 430	Taizhong1
Line 119	KinandangPatong	Line 431	Tetep （Tetepu）
Line 120	BR11	Line 432	Digu
Line 121	Huajingxian74	Line 433	C101LAC
Line 122	IR66897B	Line 434	75-1-127
Line 123	IR58025B	Line 435	CBB23
Line 124	Qb_604	Line 436	IRBB21
Line 125	Zhonghua1	Line 437	Feng986
Line 126	KCD1	Line 438	Aizaizhan
Line 127	Nionoka	Line 439	Guongchangai3784
Line 128	CYPRESS	Line 440	Qingfengai
Line 129	Shwe Thwe Yin Hyv	Line 441	Jiangerai
Line 130	PSBRC88	Line 442	Qingerai
Line 131	R106	Line 443	Jiduilun
Line 132	X23	Line 444	Kuoyedao
Line 133	C71	Line 445	Yeqinglun
Line 134	IRBB62	Line 446	Lucai
Line 135	ZH5	Line 447	Huanan15
Line 136	Jincouzhuxing	Line 448	Guangchangai4182
Line 137	NPT-100	Line 449	Guiyangai49
Line 138	EPEAL 102	Line 450	Zhaoyangzao18
Line 139	WP65	Line 451	Guichao2
Line 140	PATISAIL	Line 452	Guangchang13
Line 141	2004	Line 453	Fengqingai
Line 142	At354	Line 454	Qingnongai
Line 143	IR74	Line 455	Fengaizhan1
Line 144	1088	Line 456	Huanghuazhan
Line 145	Hua564	Line 457	Huangxinzhan
Line 146	E 2070	Line 458	28zhan
Line 147	Erjiunan1	Line 459	Fenghuazhan
Line 148	Lai nok kha	Line 460	Qingliuai1
Line 149	XIANG CHANG ZAO	Line 461	Fengbaizhan
Line 150	CHINA 1039 MUTANT(DWARF)	Line 462	Changsizhan
Line 151	Aijiaonante	Line 463	Huasizhan
Line 152	LIU LI YOU	Line 464	Yangdao2
Line 153	RONG DAO 4	Line 465	Yangdao6
Line 154	LUO AI ZAO 3	Line 466	Teqing
Line 155	M 6034-1	Line 467	Sadu-cho

Line 156	KWANG-LU-AI 4	Line 468	Shan-Huang Zhan
Line 157	E ZI 32	Line 469	IR64
Line 158	JinnanteB	Line 470	N22
Line 159	Sanbaili	Line 471	Aijiaonante
Line 160	HUO QING	Line 472	Guangluai4
Line 161	L 301B	Line 473	Xiangaizao10
Line 162	SXC216	Line 474	Qingsiai16B
Line 163	Yuenanzaodao	Line 475	Dianrui409B
Line 164	Chaoyang1B	Line 476	88B
Line 165	Jinnante43B	Line 477	Xianghui91269
Line 166	Jiangnongzao1B	Line 478	Lucai
Line 167	AN FU ZHAN	Line 479	Shufeng101
Line 168	ZhuzhenB	Line 480	Chengduai3
Line 169	Zaoxian240	Line 481	Sankecun
Line 170	Baoxie-7B	Line 482	Jiabala
Line 171	LONG GE 33	Line 483	Taishannuo
Line 172	Gzhenshan97B	Line 484	Guichao2
Line 173	KANGRI	Line 485	Luke3
Line 174	Xiangzaoxiang7	Line 486	Teqingxuanhui
Line 175	Aus/boro	Line 487	Huangsiguizhan
Line 176	Zaoshunonghu6	Line 488	Xiangwanxian3
Line 177	DECHANGBYEO	Line 489	Jinyou1
Line 178	Lucai	Line 490	Chengnongshuijing
Line 179	Qiuqianbai	Line 491	Momi
Line 180	AIJIAONANTE	Line 492	Sanbaili
Line 181	IR 9747 SEL	Line 493	Liusha1
Line 182	HONG YANG ZAO 3	Line 494	Dongtingwanxian
Line 183	Dongtingwanxian	Line 495	Yangdao2
Line 184	Shufeng101	Line 496	Liushizao
Line 185	XiangaiB	Line 497	Taizhongxianxuan2
Line 186	ZHAO DAN AI	Line 498	Nante
Line 187	Zhenfu8	Line 499	Heidu4
Line 188	Chenwan3	Line 500	Jinnante43B
Line 189	Xiangwanxian1	Line 501	Xiangzaoxiang7
Line 190	KAMULI	Line 502	80B
Line 191	CN44-40-7	Line 503	Baoxie123B
Line 192	SI WAN 14	Line 504	Jiangzaonong1
Line 193	CR579-1-3	Line 505	Gu154
Line 194	KABERI	Line 506	XiangaiB
Line 195	Sililanka1	Line 507	Aituogu151
Line 196	LIANG GUANG	Line 508	Menjiading2
Line 197	SHUANG BAI AI 2	Line 509	Jiefangxian
Line 198	SUNAR	Line 510	Baikehualuo
Line 199	W 398	Line 511	Liuyezhan
Line 200	Nante	Line 512	Zhenshan97

Line 201	DA GU AI 7	Line 513	Minghui63
Line 202	JIANG ER ZAO	Line 514	9311
Line 203	PJ110	Line 515	IRAT109
Line 204	GUANG XUAN LIU HAO	Line 516	Huajingxian74
Line 205	ZUIHOU	Line 517	Zihui100
Line 206	IR 77298-14-1-2-10	Line 518	Varylava
Line 207	Malaihong	Line 519	D62B
Line 208	Dianrui409B	Line 520	G46B
Line 209	531	Line 521	IR58025B
Line 210	FACAGRO 406	Line 522	Zhong9B
Line 211	QUN XUAN ZAO	Line 523	Xiang5
Line 212	IRAT 10	Line 524	Menjiagao2
Line 213	Guangluai15-1	Line 525	Exiang1
Line 214	Mamagu	Line 526	H198-yeza
Line 215	ARC 11777	Line 527	Huangxiuzhan
Line 216	FAN WU	Line 528	Shuhui527
Line 217	E ZI 110	Line 529	Shuhui288
Line 218	Xiaohonggu	Line 530	Shuhui707
Line 219	Dangyu5	Line 531	Longhui11
Line 220	78 XUAN WU	Line 532	Nanhui511
Line 221	KUNJUKUNJU	Line 533	Xianghui529
Line 222	PERUBAK LUEY	Line 534	Jinhui275
Line 223	RP20-12	Line 535	Luhui5240
Line 224	GZ 1368-5-4	Line 536	Minghui2088
Line 225	Aituogu151	Line 537	Shanhui287
Line 226	E ZI 100	Line 538	Shanhui8281
Line 227	CUN GU NUO	Line 539	R238
Line 228	ITA 117	Line 540	R727
Line 229	NCS349	Line 541	205R
Line 230	QING TAI AI	Line 542	781R
Line 231	PL 3165	Line 543	3301R
Line 232	Xugunuo	Line 544	IR24
Line 233	IR 19058-107-1	Line 545	IR1544
Line 234	IR 3839-1	Line 546	Gui630
Line 235	RATNAGIRI 45-2	Line 547	Fu36-2
Line 236	PORONG	Line 548	Nanhui511
Line 237	TAICHUNG SEN-YU 214	Line 549	Huhui17
Line 238	B 737G-KN-23-1	Line 550	Yihui1577
Line 239	IR 73571-3B-11-3-K2	Line 551	CDR22
Line 240	LUAN DAO	Line 552	Shuhui202
Line 241	Youzhan	Line 553	Shuhui881
Line 242	Chengduai3	Line 554	Mianhui725
Line 243	88B	Line 555	299R
Line 244	Nanjing11	Line 556	Shuhui162
Line 245	Zhenxian232	Line 557	Chenghui727

Line 246	Liusha1	Line 558	Lehui188
Line 247	BuleidaA-75	Line 559	Xinong1R
Line 248	Wanhong1	Line 560	Jinhui10
Line 249	JIN HUA 258	Line 561	Jinhui18
Line 250	KA GIL	Line 562	Jinhui16
Line 251	AR 133	Line 563	Jinhui34
Line 252	HSINCHU AI CHIO CHIENG	Line 564	Chuanhuihang907
Line 253	TAIPEI 167	Line 565	Yahui2115
Line 254	IR 77186-122-2-2-3	Line 566	Jinhui35
Line 255	Jiabala	Line 567	Wanhui88
Line 256	Teqingxuanhui	Line 568	Wanhui66
Line 257	JWR 221	Line 569	Fuhui802
Line 258	Huangsiguizhan	Line 570	Huhui602
Line 259	Gongju73	Line 571	Chenghui881
Line 260	Luke3	Line 572	Kehui746
Line 261	BOUAKE 189	Line 573	Yihui3551
Line 262	PDR 34-2-1-2	Line 574	Fuhui838
Line 263	NAN-ERH-AI 5	Line 575	Minhui3301
Line 264	SI CHAO 1	Line 576	Yihui72
Line 265	TE SAN AI 2	Line 577	Gui553
Line 266	ALTAMIRA 9	Line 578	Chenghui3203
Line 267	C 166-135	Line 579	Ehui108
Line 268	IR 63295-AC 209-7	Line 580	Huarun2
Line 269	IR 5657-33-2	Line 581	Ganghui988
Line 270	TOS 9795	Line 582	Huazhan
Line 271	K 24	Line 583	Wusansimiao
Line 272	13946(GUANG-QIU 15)	Line 584	E1573
Line 273	RP 1570-44-1	Line 585	R-Zh
Line 274	Guichao2	Line 586	S112xuan
Line 275	3210	Line 587	Ewan17B
Line 276	CN1067	Line 588	JAMBALI
Line 277	IR 10120-7-2-1-4	Line 589	-
Line 278	MBEIMBEIHUN	Line 590	DORELLA
Line 279	MURGI BRINJ	Line 591	Yanghei3
Line 280	ZACATEPEC	Line 592	Zixiang3
Line 281	71011	Line 593	Hongxiang2
Line 282	TAISEN GLUTINOUS YU 1157	Line 594	Lvxiang3
Line 283	FEDEARROZ 50	Line 595	Huangxiang2
Line 284	Kui630	Line 596	Hunanningxiangyuanzinuo
Line 285	Taizhong65/TaizhongHR539	Line 597	Hunanningxiangchangzinuo
Line 286	PR106	Line 598	Guangximomi
Line 287	Padi Ladang Ase Polo Komek	Line 599	Niboerhuangdao
Line 288	Chengnongshuijing	Line 600	DYhongdao
Line 289	LUBUK LINGGAU	Line 601	DYlvdao
Line 290	Taizhongxianxuan2	Line 602	DYheidao

Line 291	Meihuanuo	Line 603	Yunnanwenshanheinuomi
Line 292	Muguanuo	Line 604	Jiangxishangraozixiangmi
Line 293	ARC 7425	Line 605	Guangxishanglinhongxiangmi
Line 294	Xuanenchangtanqingzhan	Line 606	Fujianyouxiheiguomi
Line 295	Baikehanhe	Line 607	Fujianyouxihongguomi
Line 296	HSIEH-DAU	Line 608	Yuenanhongmi
Line 297	SPR7284-57-5	Line 609	Luhui317
Line 298	AUS 449	Line 610	15HP58
Line 299	Xianghui91269	Line 611	Luohui69
Line 300	R 762	Line 612	Zaohui43
Line 301	SHWEWARHNAN	Line 613	Zaohui49
Line 302	BAI MANG AI ZHONG	Line 614	R9519
Line 303	Jing87-304	Line 615	R5431
Line 304	EX MARABA-GURUKU	Line 616	99-14
Line 305	Haogelao	Line 617	900
Line 306	IR 28	Line 618	Feng9
Line 307	C418	Line 619	1128
Line 308	KURULU WEE(WHITE)	Line 620	9348
Line 309	FIDJI	Line 621	-
Line 310	TAK SUFAID	Line 622	Xianghongjiangxifuzhoulinchuuan
Line 311	SLAVA	Line 623	Zimidaojiangxifuzhoulinchuan
Line 312	SAHEL 108		

Grey filling materials come from 3K RGP, in total of 323 varieties .
